# Effects of Surface Treatment Conditions on the Bonding Strength and Electromagnetic Pulse Shielding of Concrete Using the 85Zn-15Al Arc Thermal Metal Spraying Method

**DOI:** 10.3390/ma16041372

**Published:** 2023-02-06

**Authors:** Jongmin Jang, Kwangwoo Wi, Han-Seung Lee, Jitendra Kumar Singh, Han-Hee Lee

**Affiliations:** 1 Department of Smart-City Engineering, Hanyang University, 1271 Sa 3-dong, Sangrok-gu, Ansan 15588, Republic of Korea; 2 Innovative Durable Building and Infrastructure Research Center, Hanyang University, 1271 Sa 3-dong, Sangrok-gu, Ansan 15588, Republic of Korea; 3 Department of Architectural Engineering, Hanyang University, 1271 Sa 3-dong, Sangrok-gu, Ansan 15588, Republic of Korea; 4 Innovative Durable Building and Infrastructure Research Center, Center for Creative Convergence Education, Hanyang University, 1271 Sa-3-dong, Sangnok-gu, Ansan 15588, Republic of Korea; 5 Department of Chemistry, Graphic Era Deemed to be University, Bell Road, Clement Town, Dehradun 248002, Uttarakhand, India; 6 Korea Testing Laboratory, 723 Haean-ro, Sangnok-gu, Ansan 15588, Republic of Korea

**Keywords:** concrete, electromagnetic pulse shielding, arc thermal metal spray, bonding strength, shielding effect

## Abstract

The surface treatment of concrete enhances the bonding of its metal coatings. Therefore, in the present study, on the concrete surface, prior to the deposit of an 85Zn-15Al coating via an arc thermal spraying process, different surface treatments were considered for the effective electromagnetic pulse (EMP) shielding properties of the concrete. However, the direct coating on a concrete surface possesses lower bond adhesion, therefore it is of the utmost importance to treat the concrete surface prior to the deposition of the metal coating. Moreover, to obtain better bond adhesion and fill the defects of the coating, the concrete surface is treated by applying a surface hardener (SH), as well as a surface roughening agent (SRA) and a sealing agent (SA), respectively. The metal spraying efficiency, adhesion performance, and bonding strength under different concrete surface treatment conditions were evaluated. The EMP shielding effect was evaluated under the optimal surface treatment condition. The proposed method for EMP shielding exhibited over 60% of spraying efficiency on the treated surface and a bonding strength of up to 3.9 MPa for the SH–SRA–SA (combining surface roughening and pores/defects filling agents) specimen compared to the control one, i.e., 0.8 MPa. The EMP shielding values of the surface-treated concrete with surface hardener, surface roughening agent, and sealing agent, i.e., SH–SRA–SA specimens, exhibited 96.6 dB at 1000 MHz. This was about 12 times higher than without coated concrete.

## 1. Introduction

Electromagnetic pulses (EMP) of 50–100 kV/m that are released naturally or by nuclear weapons can cause social havoc and significant damages to major national infrastructures, such as communication networks, electronic equipment, and water systems [1]. EMPs do not directly harm humans but may instantly paralyze major national facilities through circuitry breakdown by inducing an overcurrent via the generation of an intense electromagnetic induction in the electronic device circuits [1,2,3,4]. Therefore, facilities must be protected against EMP damage. The concrete used in buildings has an extremely high electrical resistivity of 10^6^–10^9^ Ω·m [5,6], rendering it vulnerable to EMP [7,8]. To minimize EMP damage, materials with low electrical resistivity, such as copper (1.7 × 10^−8^ Ω·m), are employed in concrete buildings [9,10].

When an EMP is transmitted via a highly conductive substance, reflection and absorption repeatedly occur, which reduce the intensity of or eliminate the EMP—a process referred to as EMP shielding. This shielding effect is influenced by the electrical conductivity, permeability, and thickness of the material used [11,12]. Metal panels, which are typically used for shielding, are bolted or welded onto existing concrete structures. However, this increases the construction cost, while the space and construction efficiency decrease when a new room is added to the existing concrete structure. Moreover, the corrosion of welds or joints produces oxides that reduce the shielding efficacy [13,14].

At 1 GHz, materials with high electrical conductivity, such as carbon black, carbon nanotubes, and steel fibers, can be incorporated into concrete to improve the shielding effect of the concrete structure [14,15,16,17,18,19,20]. However, the mass production of these materials is high-cost and their economic efficiency is low. Furthermore, their addition to concrete requires a water-reducing agent or an increase in the unit quantity of the materials to ensure workability, which reduces economic efficiency and the strength of the concrete, respectively [10,20].

The arc thermal metal spraying method is used to improve the corrosion and EMP resistance of the structures. In this process, an electric arc heat is generated by the current flowing between two metal wires. The metal sprayed at the arc point on the substrate cools and travels through air before colliding with the surface and solidifying, which laminates the coating. Therefore, metal spraying is performed after the treatment of the steel surface. On the other hand, the use of concrete, as low-conducting material, means it cannot be used alone for EMP shielding. To increase the conductivity of the concrete, materials such as carbon black, graphite, and MXene are used, owing to their exceptional electrical conductivity, hydrophilicity, and chemical activity [16,21,22,23,24]. However, there are other methods to produce high EMP efficiency, but these are expensive. Although carbon nanofiber [22] or copper can be used in concrete, nanofiber is expensive and difficult to mix into concrete. The cladding needs a thick layer of metal plate, where the cost is increased [25]. These materials are very expensive, therefore, it is suggested to use a sealing agent on the concrete surface before the spray-laminated porous metal coating [26,27].

Owing to the high corrosion resistance of metal spray coatings, researchers have performed experiments to assess the durability of steel and concrete structures subjected to arc thermal metal spraying. The EMP shielding capability of non-electrically conductive concrete was investigated using the features of highly conductive metallic coatings [28,29]. A previous study on the EMP shielding effect indicated that a shielding magnitude exceeding 80 dB, as stipulated in MIL-STD-188-125-1 [30], could be achieved by coating the concrete surface with different metals and thicknesses of several hundred micrometers [29]. However, thermally sprayed metal particles do not make proper bonding at the coating–concrete interface, owing to an inadequate surface roughness while being laminated on the concrete surface. Thus, surface treatment methods must be reviewed to ensure their constructability and appropriate adhesion.

As described above, concrete possesses extremely high electrical resistivity, therefore, metal spray coating is used on it to increase its conductivity. The direct deposition of the metal coating via the arc metal thermal spray process exhibit lower bond adhesion to be considered for an EMP. There is weak interface zone between the concrete and the metal coating, therefore, a surface roughening agent is required to make them strongly bonded. Epoxy-modified cement-based material is frequently used as a roughening agent [31]. Moreover, asphalt-modified sulfoaluminate cement exhibits less bonding with concrete [32].

Therefore, in the present study, we have treated the concrete surface prior to the deposition of the 85Zn-15Al coating and applied the pores/defects filling agent to reduce the porosity of the coating. Two different concrete surface treatments, such as surface hardener (SH) and surface roughening agent (SRA), were used to increase the adhesion of the 85Zn-15Al coating with the concrete, thereafter a pores/defects sealing agent (SA) was applied over the coating surface to study the deposition efficiency, bonding strength, and EMP shielding effect of concrete applied via the arc thermal metal spraying method. Subsequently, an arc thermal metal spraying method with proper surface treatments is proposed to ensure the effective EMP shielding performance of the concrete.

## 2. Methods and Materials

### 2.1. Experimental Overview

The metal spraying efficiency and bonding strength were determined based on the surface treatment conditions of the concrete and metal spray coating. This enables the application of the arc thermal metal spraying method to concrete for EMP shielding (see Table 1). To increase the surface roughness of the concrete for proper bonding, two different types of agent, i.e., surface hardener (SH) and surface roughness agent (SRA), were used (Table 1). The SH, i.e., an odorless and colorless lithium-based resin, was applied with a nylon brush, whereas the SRA, i.e., silica sand of 0.18 to 0.25 mm size with an epoxide resin (polyepoxides) was applied via spray process, i.e., a gun on the hardened concrete to induce the mechanical interlocking with the concrete surface to fill the irregularities/pores or defects of the deposited 85Zn-15Al coating by spraying the epoxide resin. The details are explained in Table 1. A concrete mold (300 mm × 300 mm × 100 mm) was treated according to the experimental factors to assess the metal spraying efficiency. The adhesion performance was assessed based on the concrete surface treatment conditions and the presence/absence of a metal coating sealing treatment. The EMP shielding effect of a 200 μm thick metal sprayed coating on the concrete surface with different roughening and sealing agents was assessed [29].

### 2.2. Arc Thermal Metal Spraying Method

**Figure 1:**
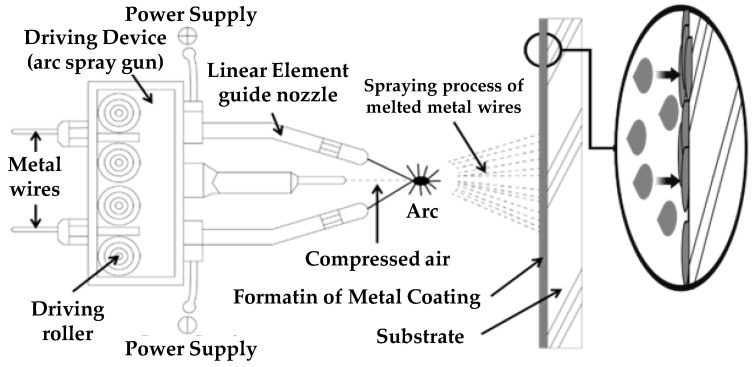
Schematic of arc thermal metal spray system.

Figure 1 shows the arc thermal metal spraying method. The metal was melted by an arc heat source generated by a high-voltage current between electrodes, using a thermal spray material as the consumption electrode. Spraying was performed with compressed air to apply the coating. Different metals are utilized in various industrial fields to increase the abrasion resistance, heat resistance, and corrosion resistance. The two wires that were oppositely charged were melted at the arc point where the molten metal particles were sprayed with compressed air, cooled in the air, and then deposited on the coating. The coating film forms a layer and hardens after it collides with the underlying substrate. Because the particles collide with the substrate in the cooled condition to form a coating layer, no thermal deformation occurs on the substrate. However, the thermal spray coating causes porosity defects. Previous studies on this porous metal spraying method have shown that thermally sprayed coatings with a thickness of 100 μm are unsuitable for EMP shielding, owing to defects within the coating layer [29].

### 2.3. Fabrication of the Concrete Specimen

**Figure 2:**
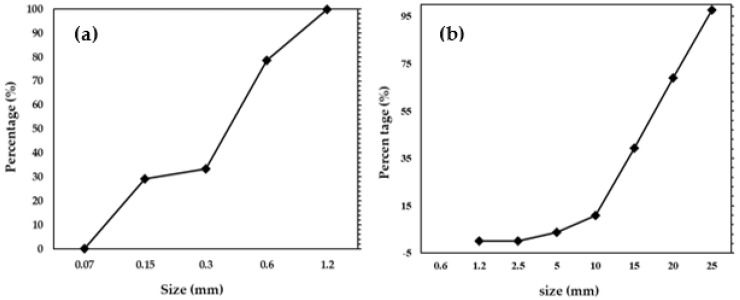
Particle size distribution of (**a**) fine and (**b**) coarse aggregates.

For the fabrication of the concrete, the water-to-cement ratio was kept at 50% using type 1 ordinary Portland cement (OPC), i.e., KS L 5201 [33], and coarse and fine aggregates of up to 25 mm and 5 mm in size, respectively. The physical properties and chemical composition of OPC is shown in Table 2. The particle size distribution of fine and coarse aggregates is shown in Figure 2a,b, respectively. A polycarboxylate water-reducing agent with 0.8% cement weight was added to ensure fluidity. The physical properties, i.e., the slump test, unit volume mass, and air measurement of the unhardened concrete were tested in accordance with KS F 2402 [34] and KS F 2409 [35]. The concrete was poured into a 300 mm × 300 mm × 100 mm mold, and then cured at 20 ± 2 °C and a relative humidity of 60 ± 2% for 1 day. Thereafter, it was kept for 27 days of water curing. The compressive strength of the concrete was measured by a 30T class universal testing machine (DEC-30TC, DA-WHA, Bucheon-si, Gyeonggi-do, Republic of Korea) at a 2400 N/s loading rate. Table 3 lists the proportions of mixtures utilized in this study to fabricate the concrete specimen.

### 2.4. Test Methods

The metal spray coating’s thickness, morphology, efficiency, bonding strength, and EMP shielding effect were assessed based on the surface treatment methods to evaluate the mechanical properties of the arc thermal metal sprayed concrete and metal coating.

#### 2.4.1. Metal Spraying Thickness and Efficiency

The 85Zn-15Al coating thickness was measured by a non-destructive Elcometer456 (Tokyo, Japan) at four different locations and their averages are reported in the manuscript.

The deposition efficiency of the coatings was determined based on ISO 17836 [36] as follows:

*η_D_* = Δ*m_tp_*/Δ*m_sm_* × 100%, (1)

where *η_D_* is the metal deposition efficiency (%), Δ*m_tp_* is the weight difference of the specimen (g), and Δ*m_sm_* is the weight difference of the sprayed material (g).

The metal spraying efficiency was calculated by multiplying the weight difference between the specimens before and after thermal spraying by the weight difference between the thermally sprayed materials. This reflects the efficiency in depositing thermally sprayed metal particles using different treatment methods.

#### 2.4.2. Bonding Strength

**Figure 3:**
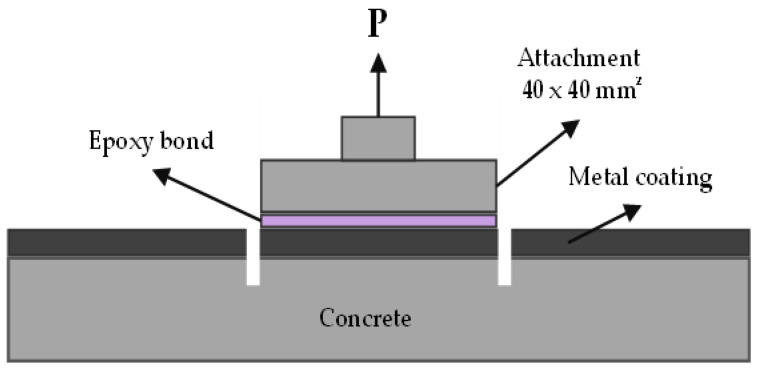
Diagram of bonding strength measurement.

To assess the bonding strength between the thermally sprayed coating and the concrete, an epoxy resin was used to bind a 40 mm × 40 mm square knob at nine different locations in accordance with KS F 4716 [37]. After 24 h of drying the epoxy resin, a groove was cut into the substrate; a tensile force of 1750 ± 250 N/min was applied in the vertical direction, and the bonding strength was measured by dividing the maximum tensile load by the attachment area. The measurement methods are shown in Figure 3. The interface failed at the weakest location during the bonding strength test. The failure patterns were classified as interfacial or non-interfacial failures, depending on the failure locations. An interfacial failure occurred at the interface of the concrete and the coating. When the bonding strength between the concrete and the coating exceeded the strength of the concrete, a non-interface failure occurred. The interfacial failure pattern was characterized in this study based on the surface treatment conditions. The non-interfacial failure area of the concrete was estimated using the ImageJ software (version 1.52n)( NIH, Bethesda, MD, USA). The non-interfacial failure area of the concrete was determined using the ImageJ software after it was converted into a grayscale image.

Bonding strength (MPa) = T/A(2)

where T is the maximum tensile load (N) and A is the area (1600 mm^2^).

#### 2.4.3. Surface Morphology

The coating surface morphology and cross section of the control specimen was determined by a field emission scanning electron microscope (FE-SEM, MIRA3, TESCAN, Brno, Czech Republic) at 15 kV. The porosity of the coating was measured by the open source ImageJ software (NIH, Bethesda, MD, USA) ImageJ software (version 1.52n) [38,39,40].

#### 2.4.4. EMP Shielding Effect

**Figure 4:**
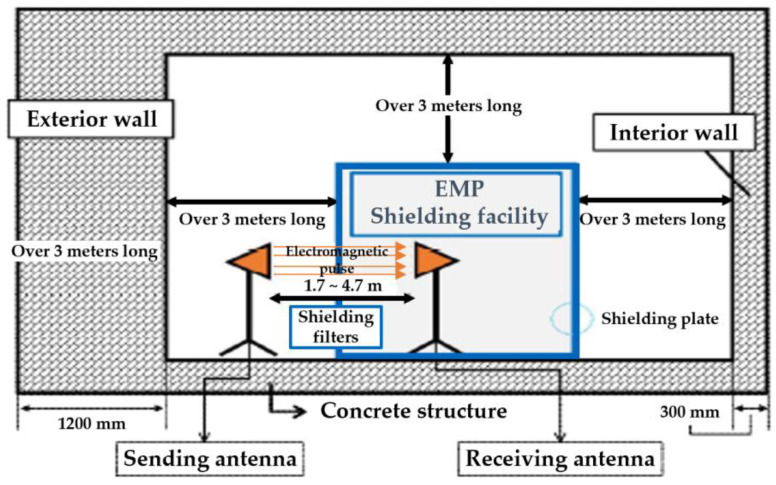
Concrete specimen shielding effect test.

The EMP shielding effect of the concrete, as well as different surface treatment specimens coated via the arc thermal metal spray process were assessed based on MIL-STD-188-125-1 (high-altitude EMP protection for fixed ground-based facilities) [30]. Figure 4 shows the environment for assessing the EMP shielding effect on the specimen. The metal spray-coated concrete specimen was mounted at the opening of the metal-shielding room. The internal and external antennas that radiated EMPs were spaced 3 m apart. The magnitude of the power transmitted through the metal spray-coated concrete was measured to assess the EMP shielding effect. The shielding effect was quantified using the S-parameter formula, which is expressed as the ratio of the transmitted signal (V_0_) and the received signal (V_REV_) as follows:

SE_metalcoating_ = 20 log(V_0_/V_REV_) (3)

## 3. Results and Discussion

### 3.1. Metal Spray Morphology and Efficiency

**Figure 5:**
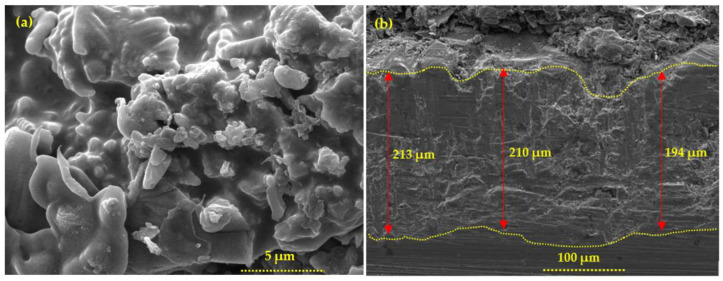
SEM of (**a**) top surface at 10,000× and (**b**) cross section at 500×.

A 200 µm thick 85Zn-15Al coating was considered to study the EMP shielding properties. Initially, the coating thickness was measured by an Elcometer456 and found to be 200 (±10) µm. The surface morphology of the coating is shown in Figure 5. Figure 5a shows the top surface morphology of the coating where splat and coarse particles with some defects are deposited. This coating is dense and exhibited some inflight particles caused by the cooling of molten metal particles. The coating thickness is verified by the cross section, as shown in Figure 5b. The coating thickness is found to be 200 (±10) µm. This is well-corroborated by the thickness measured by the Elcometer456. The porosity of the coating was measured by the cross section images and found to be 7.55 (±0.63) %. Therefore, the SA was used to fill the defects of the deposited coating.

### 3.2. Properties of the Concrete

The physical properties of the concrete are shown in Table 4. The slump values of the concrete are found to be 180 mm while the air content is 4.3%. The compressive strength of the concrete is 29.27 MPa after 28 days of curing.

**Figure 6:**
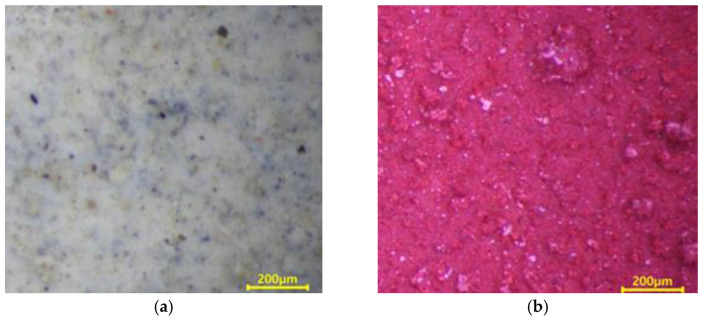
Surface images (**a**) SH and (**b**) SRA at 250×.

The metal spraying efficiency is defined in ISO 17836 [36] as the change in the weight of the concrete specimen after being subjected to the arc thermal metal spraying, divided by the wire consumption. As shown in Figure 6a, no change was detected on the concrete surface after the application of the lithium-based SH. However, when the SRA was combined with silica sand, a red epoxy coating layer formed on the entire concrete surface, as shown in Figure 6b. Simultaneously, the silica sand mixed with the epoxy coating layer protruded. These surface roughening agents increase the roughness of the concrete surface.

**Figure 7:**
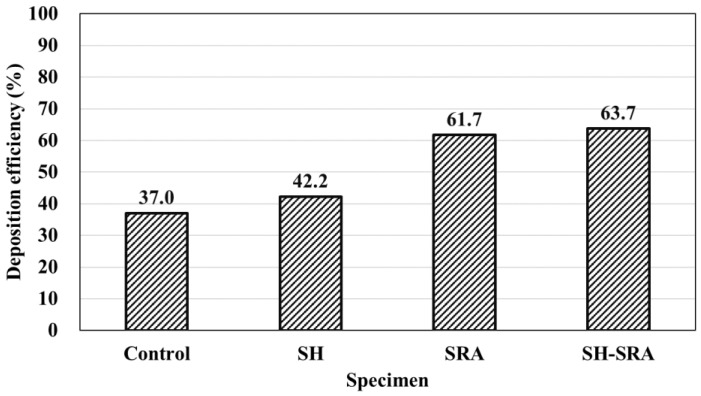
Deposition efficiency of metal spray coating according to surface treatment conditions.

Figure 7 shows the metal spraying deposition efficiency on the concrete specimens subjected to surface treatment. The metal spraying efficiency of the control specimen without surface treatment was 37%, whereas the value for the specimen with SRA and SH–SRA exceeded 60%. The metal spraying efficiency of the specimen treated with the lithium-based SH was approximately 42.2%, which is not significantly different from that of the control specimen. The metal spraying efficiencies of the SRA and SH–SRA specimens were 61.7% and 63.8%, respectively. This shows that using the SRA increased the metal spraying efficiency by approximately 70% compared with the control specimen, attributed to the proper anchoring with the concrete surface where both, i.e., SH and SHR, synergistically help to increase the deposition efficiency.

Because all the laitance on the surface of the concrete specimens was removed, the metal spraying efficiency resulting from the chemical interaction between the metal particles and the concrete could not be determined. Hence, the metal spraying efficiency was low for both the control and the SH specimens. The roughness of the SH–SRA specimen was found to be 75–220 μm, where the epoxy-based silica increased the anchoring properties of the metals with the concrete, which lead to a widening in the interfacial zone between the concrete and the roughening agent.

### 3.3. Bonding Strength of Metal Spray Coating

Table 5 shows the results of the bonding strength based on the treatment conditions. The control specimen without any surface treatment exhibited the lowest bonding strength, i.e., 0.8 MPa. The bonding strength of the concrete specimen coated with the SH was found to be 1.2 MPa, which is less than 1.5 MPa, as recommended in KS F 9001 [41]. The minimum bonding strength of the coating should be 1.5 MPa, but the control and SH specimens exhibited lower than this value. The bonding strength of the SRA-coated specimen is around four times higher than that of the control specimen. The bonding strengths of the SH–SRA and SH–SRA–SA specimens were 3.7 and 3.9 MPa, respectively. This is around 20–30% higher than the SRA specimen.

**Figure 8:**
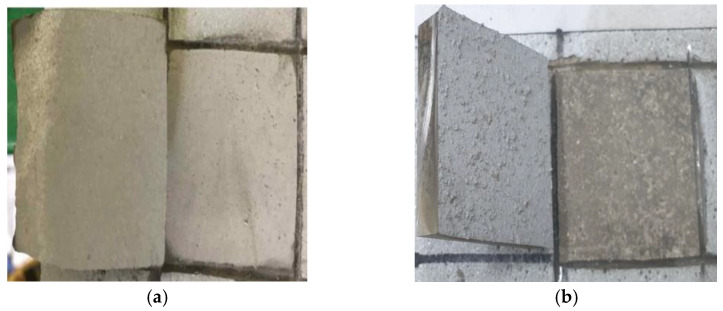
Interfacial failure mode according to surface treatment conditions. (**a**) Control; (**b**) SH.

**Figure 9:**
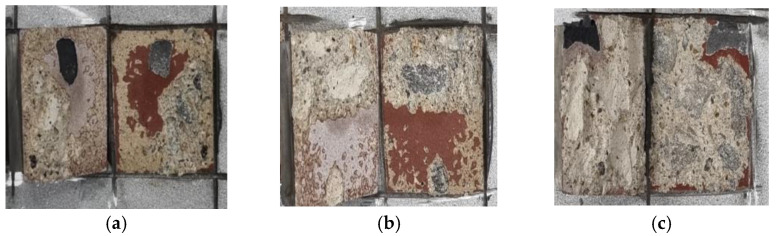
Non-interfacial failure mode according to surface treatment conditions. (**a**) SRA; (**b**) SH–SRA; (**c**) SH–SRA–SA.

Figure 8 shows the failure patterns of the control and SH specimens. Based on this figure, interfacial failure was verified for both the concrete and metal spray coating surfaces. The non-interfacial failure in the concrete component of the failed region was observed in the SRA, SH–SRA, and SH–SRA–SA specimens, as shown in Figure 9. The integrity of the concrete–metal interface was secured in the specimens with the SRA by enhancing the bonding strength between the concrete and thermally sprayed metal coating. Therefore, the physical properties of the thermally sprayed coating, arising from the mechanical interlocking of silica sand, were improved.

Table 6 presents the non-interfacial and interfacial failure areas in black and white, respectively, which were determined using an ImageJ analysis program that focused on the areas of non-interfacial failure. The ratio of non-interfacial area to total area (non-interface failure area ratio) was calculated. The results show that the non-interface failure area ratios of the SRA, SH–SRA, and SH–SRA–SA specimens were approximately 51%, 67%, and 84%, respectively, whereas the control and SH-treated specimens show extremely low. This result suggests that these two specimens exhibit failure characteristics where the interfacial zone has low anchoring properties, therefore, they show low adhesion value, which correlates with the bonding values (Table 5).

**Figure 10:**
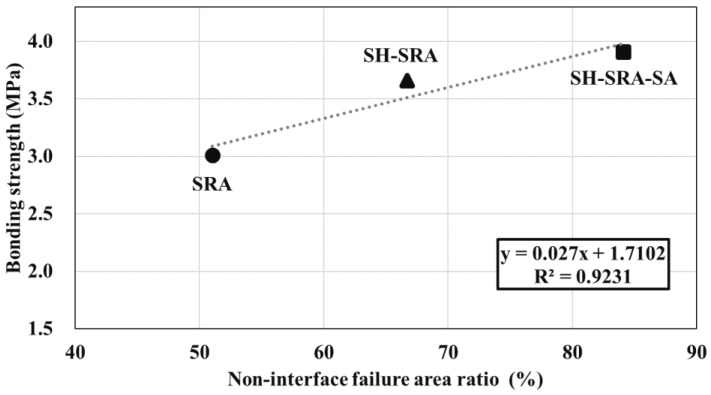
Relationship between bonding strength and non-interface failure area ratio.

Figure 10 shows the relationship between the non-interface failure area ratio and the bonding strength, i.e., the bonding strength increased with the non-interfacial area of the concrete. The SRA enhanced the weak region of the interface between the metal coating and the concrete, while increasing the non-interfacial failure area at the concrete interface. When the surface treatment is applied to the concrete surface, the non-interfacial failure area is increased with the strength of the concrete. When the sealing agent is applied, the bonding strength may have been increased by the sealing agents, i.e., epoxide penetrating through the defects/pores of the 85Zn-15Al coating layer to reach the substrate, therefore, it exhibits the highest bond adhesion and non-interfacial area.

### 3.4. EMP Shielding Effect of 85Zn-15Al Coating

**Figure 11:**
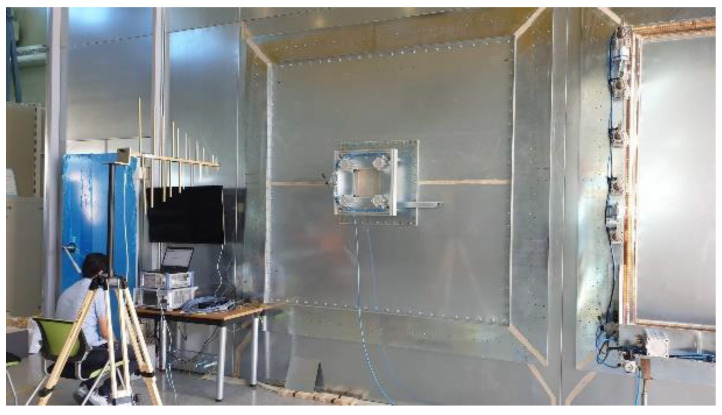
Shielding effectiveness measurement of concrete.

The EMP shielding effect of the 85Zn-15Al arc thermal metal sprayed coating on the concrete is investigated at seven frequencies: 600, 900, 1000, 1200, 1500, 1800, and 2000 MHz. The concrete specimen without the arc thermal metal spraying and surface treatment (concrete), the specimen with the thermally sprayed coating and no surface treatment (control), and the SH–SRA–SA specimen are only compared to evaluate the EMP shielding effect. The SH–SRA–SA specimen exhibited the highest metal spraying efficiency and bonding strength, whereas the concrete specimen was the substrate and the control specimen had only the 85Zn-15Al coating. The EMP shielding effect was measured (Figure 11) by mounting identical receiving antennas inside the shielding room. During the test, the V_0_ was measured based on the bare opening between the two antennas, and the V_rev_ of the concrete specimen. Next, the EMP shielding effect was determined by Equation (3).

**Figure 12:**
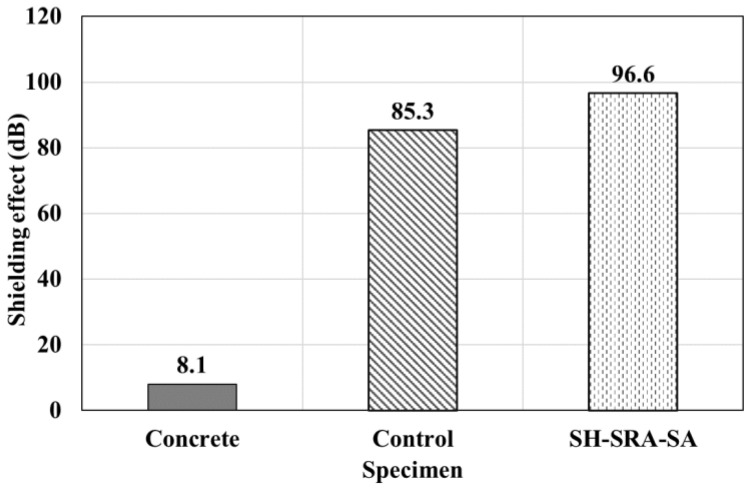
Results of shielding effect at 1000 MHz.

Figure 12 shows the EMP shielding effect for each concrete specimen at a frequency of 1000 MHz. Most national security buildings, military base camps and hospitals need to be protected by electromagnetic interference at 1000 MHz, where the minimum EMP shielding value must be 80 dB [30]. Therefore, it is compared with only a particular frequency. The EMP shielding effect of concrete without the arc thermal metal spraying is less than 10 dB. In contrast, the performance of the control and SH–SRA–SA specimens coated with the 85Zn-15Al metal with a thickness of 200 µm, via arc thermal metal spraying, is higher than 80 dB. This suggests that EMP shielding can be achieved using the arc thermal metal spraying method, regardless of the physical properties between the concrete and metal coating.

**Figure 13:**
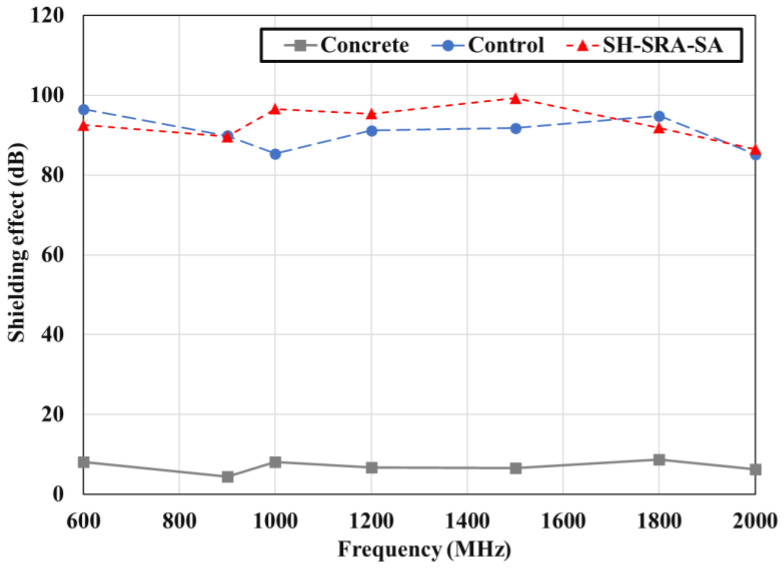
Results of shielding effect of concrete.

Figure 13 shows the frequency-dependent variations in the EMP shielding effect of the concrete specimens. At each frequency, the EMP shielding effect of the concrete specimen without the arc thermal metal spraying is less than 10 dB, indicating that this effect could not be achieved using only concrete materials. In contrast, the control and SH–SRA–SA specimens exhibited a shielding effect of over 80 dB across all frequency bands. This result is similar to that observed at 1000 MHz. However, between 1000 and 1500 MHz, the SH–SRA–SA and control specimens exhibited different shielding effects. These differences are attributed to the difference in the physical properties of the metal coating and the concrete, as confirmed by the deposition efficiency and bonding strength.

## 4. Conclusions

In this study, the concrete specimens were coated with 85Zn-15Al via an arc thermal metal spraying process, as well as applied with different treatments on the concrete and coating. The effects of the surface treatment conditions and metal spray coating on the deposition efficiency, bonding strength, and EMP shielding effect were quantitatively analyzed. The following conclusions were drawn:

1. The deposition efficiencies of SRA and SH–SRA were approximately 60% and 70% higher than those of the control specimen without the SRA, respectively.
2. The bonding strength of the metal spray coating was 3.7 MPa after the SH and SRA were used to reinforce and create roughness on the concrete surface, respectively, which is four times greater than that of the concrete without any surface treatment. The usage of the SRA was particularly effective in preventing the interfacial failure of the metal spray coating. In addition, by applying the SA, the bonding strength was improved due to the penetration of the SA into the layer of the concrete.
3. The EMP shielding value of the concrete was less than 10 dB. However, when the 85Zn-15Al coating was applied to the concrete surface, the EMP shielding value reached 80 dB or higher. This confirms that the spraying of the 200 µm thick 85Zn-15Al coating on the surface of the concrete structure provides an EMP shielding value of greater than 80 dB. This coating could be used in protecting a national security building, military base camp or hospital from EMP effects.
4. The surface treatment of the concrete and coating enhances the properties of the specimen. The SH had less influence in deposition efficiency and bonding, whereas once the SRA and SRA, along with the SA, were applied, the specimen exhibited the highest bonding and EMP value. Where the SRA helped in making the concrete surface rougher, owing to the epoxy and silica sand, the SA helped in the filling of the defects of the 85Zn-15Zn coating.

## Figures and Tables

**Table 1:** Experimental variables.

No.	Specimen Name	Surface Hardener	Surface Roughness Agent	Metal Spray	Sealing Agent
1	Control	X	X	O	X
2	SH	O	X	O	X
3	SRA	X	O	O	X
4	SH–SRA	O	O	O	X
5	SH–SRA–SA	O	O	O	O

SH: Surface Hardener, SRA: Surface Roughness Agent, SA: Sealing Agent.

**Table 2:** Chemical composition and properties of OPC used in present study.

	Specific Surface Area (cm^2^/g)	Density (g/cm^3^)	Chemical Compositions (%)						
			SiO_2_	Al_2_O_3_	Fe_2_O_3_	CaO	MgO	SiO_3_	Others
OPC	3412	3.14	20.57	5.48	3.18	63.03	3.41	2.12	2.20

**Table 3:** Mix proportion of concrete used in this study.

W/C (%)	S/a (%)	Air (%)	Unit Weight (kg/m^3^)				Admixture (%)
			Water	Cement	Sand	Gravel	
50	52	4.5	175	350	905	835	0.8

**Table 4:** Physical properties of the concrete.

Slump Value (mm)	Air Content (%)	Compressive Strength (MPa)
180	4.3	29.27

**Table 5:** Results of bonding strength.

Specimen Name	Bonding Strength (MPa)										SD	CV
	1	2	3	4	5	6	7	8	9	Ave.		
Control	0.6	0.7	0.8	0.8	0.6	0.6	0.9	0.8	1.0	0.8	0.2	0.2
SH	1.1	1.2	1.3	0.8	1.1	1.4	1.2	0.6	2.1	1.2	0.4	0.4
SRA	2.2	3.6	2.9	2.8	4.1	3.1	2.8	3.2	2.5	3.0	0.6	0.2
SH–SRA	3.5	3.8	3.9	3.5	3.4	3.7	3.6	3.6	3.8	3.7	0.2	0.0
SH–SRA–SA	3.8	3.7	3.9	4.0	3.9	4.0	4.0	4.2	3.8	3.9	0.2	0.0

SH: Surface Hardener, SRA: Surface Roughness Agent, SA: Sealing Agent.

**Table 6:** Non-interface failure area and ratio according to face treatment conditions.

	Control	SH	SRA	SH–SRA	SH–SRA–SA
Non-interface failure area	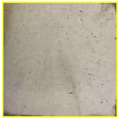	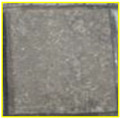	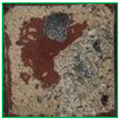	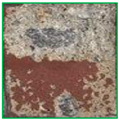	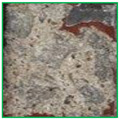
Non-interface failure area in black and white	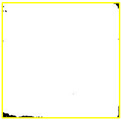	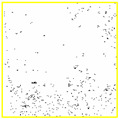	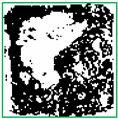	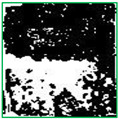	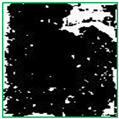
Non-interface failure area ratio	0.5%	1.5%	51.0%	66.7%	84.1%

## Data Availability

Not applicable.
